# Amino Acid-Driven Mitochondrial Metabolic Rewiring Controls Antitumor Immunity

**DOI:** 10.3390/cancers18091474

**Published:** 2026-05-03

**Authors:** Suji Ham, Min-Jeong Jo, Kwon-Ho Song, Bo-Hyun Choi

**Affiliations:** 1 Department of Pharmacology, School of Medicine, Daegu Catholic University, Daegu 42472, Republic of Korea; sjham@cu.ac.kr (S.H.); cmjdgt0423@cu.ac.kr (M.-J.J.); 2 Department of Cell Biology, School of Medicine, Daegu Catholic University, Daegu 42472, Republic of Korea; khsong@cu.ac.kr

**Keywords:** tumor microenvironment, mitochondrial metabolism, amino acid metabolism, antitumor immunity, immunotherapy resistance

## Abstract

Amino acids are essential nutrients for both tumor growth and immune cell function. Cancer cells actively deplete intracellular and extracellular amino acid pools, and limited amino acid availability in the tumor microenvironment (TME) reinforces immunosuppression. Mitochondria are not merely adenosine triphosphate-producing organelles. Amino acid metabolism within mitochondria contributes to tumor progression and influences immune cell fate and effector function. These effects are mediated through biosynthetic precursor generation for lipid, nucleotide, and polyamine synthesis, maintenance redox homeostasis through glutathione and NAD^+^ metabolism, and regulation of gene expression through aryl hydrocarbon receptor signaling. In this review, we discuss four major mitochondrial amino acid metabolic pathways: glutamine-driven anaplerosis, serine/glycine-dependent one-carbon metabolism, arginine–ornithine metabolism, and tryptophan–kynurenine metabolism. We examine how these pathways are rewired in cancer cells, how they influence immune cell function through direct or mitochondria-associated mechanisms, and how such metabolic reprogramming promotes tumor progression while impairing antitumor immunity. Finally, we consider therapeutic strategies to improve cancer immunotherapy by targeting amino acid metabolism, including mitochondrial metabolic enzymes. This review may help guide the development of more effective metabolic biomarkers and mitochondria-based therapeutic strategies for cancer immunotherapy.

## Simple Summary

Cancer cells and immune cells both rely on amino acids to survive and function. In tumors, however, amino acid use is often rewired to support cancer growth while weakening the body’s antitumor immune response. Mitochondria, widely known for producing cellular energy, are also important sites of amino acid metabolism. This review examines four major amino acid pathways and explains how mitochondrial metabolic rewiring enables tumor cells to adapt and grow, while simultaneously altering immune cell function. It further discusses how these pathways might be targeted to improve cancer treatment. By integrating these findings, this review provides a clearer framework for understanding how mitochondrial amino acid metabolism shapes both tumor progression and immune dysfunction. It also highlights its potential for developing more precise biomarkers and treatment strategies.

## 1. Introduction

Amino acids are essential metabolic substrates for both tumor cells and immune cells [1,2]. In cancer, the demand for amino acids is profoundly increased because rapidly proliferating tumor cells require sustained supplies of carbon and nitrogen to support growth, survival, and adaptation to environmental stress [1,3]. Cancer cells actively consume and redistribute amino acid pools within the tumor microenvironment (TME), creating a metabolically restrictive niche that constrains the activation, differentiation, and effector function of tumor-infiltrating immune cells [1,2,4]. Accordingly, amino acid metabolism has been widely investigated as both a determinant of tumor progression, and a therapeutic vulnerability in cancer immunotherapy [1,2,5,6]. However, prior studies and reviews have primarily emphasized nutrient availability and competition in the TME. It remains less clearly understood how amino acid-dependent metabolic programs converge on mitochondrial function across tumor and immune compartments, and how this convergence influences antitumor immunity and therapeutic responsiveness [5,6].

Amino acid metabolism takes place not only in the cytosol but also in the mitochondria [7]. Glutamine is extensively metabolized in mitochondria, where it serves as a major carbon source for tricarboxylic acid (TCA) cycle anaplerosis, following glucose [8,9]. Serine is synthesized *de novo* in the cytosol, whereas much of its downstream catabolism that supports biosynthetic precursor generation occurs in mitochondria. In this compartment, serine is converted to glycine by *SHMT2*, fueling one-carbon metabolism for nucleotide synthesis and supporting redox homeostasis [10,11]. In addition, part of arginine metabolism, including the urea cycle, occurs in mitochondria [12], and components of tryptophan metabolism are catalyzed by enzymes localized to the mitochondrial membrane [13]. Across these pathways, TCA cycle-driven ATP production, biosynthesis precursor generation, and glutathione- and NAD^+^-linked redox homeostasis. These functions collectively play essential roles in both tumor progression and immune cell function [2,8,10]. Thus, mitochondria should be regarded not simply as ATP-producing organelles, but as a central regulatory hub integrating amino acid metabolism with central function.

Amino acid depletion in the TME and the resulting metabolic adaptation of immune cells may perturb mitochondria-associated amino acid metabolism, thereby compromising antitumor immunity across multiple immune cell types [14,15,16]. Collectively, these changes impair T-cell effector function, promote exhaustion-associated states, reduce NK-cell cytotoxicity, and reinforce immunosuppressive programs in myeloid cells [17,18,19,20]. Such metabolic constraints may partially explain the limited efficacy of immune checkpoint inhibitors (ICIs) in many tumors [21].

**Figure 1:**
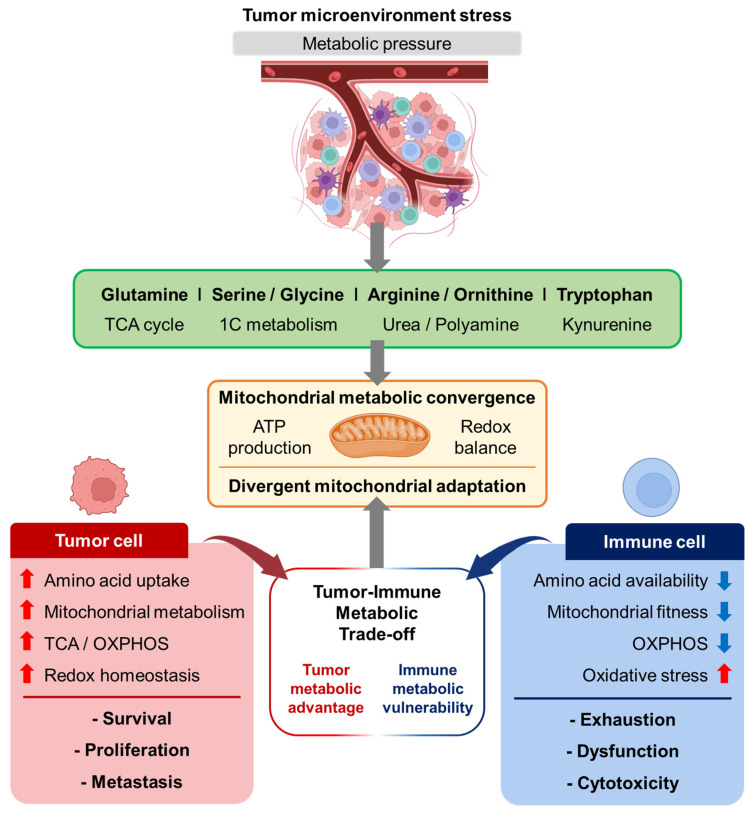
Mitochondrial metabolic convergence and divergent adaptation in tumor and immune cells under tumor microenvironment (TME). Metabolic pressure in the tumor microenvironment (TME) drives the coordinated utilization of key amino acid pathways—including glutamine (TCA cycle), serine/glycine (one-carbon metabolism), arginine/ornithine (urea cycle and polyamine synthesis), and tryptophan (kynurenine pathway)—which converge on mitochondrial functions such as ATP production and redox balance. Despite this shared metabolic convergence, tumor and immune cells undergo divergent adaptations: tumor cells enhance amino acid uptake, mitochondrial metabolism, and OXPHOS to sustain redox homeostasis, thereby promoting survival, proliferation, and metastasis, whereas immune cells experience reduced nutrient availability, impaired mitochondrial fitness (ATP production, redox balance, and metabolic flexibility), and increased oxidative stress, leading to functional exhaustion and diminished cytotoxic activity. The arrows show the direction of the process: metabolic pressure in the tumor microenvironment leads to activation or involvement of amino acid pathways, which then converge on mitochondrial metabolism and result in different adaptations in tumor cells and immune cells. The upward arrows mean that a feature is increased, and the downward arrows mean that a feature is decreased.

These observations support a “tumor–immune metabolic trade-off” within the TME, in which tumor and immune cells compete for limited amino acid resources but exhibit fundamentally asymmetric metabolic outcomes. Tumor cells gain a metabolic advantage by enhancing amino acid uptake, mitochondrial rewiring, and metabolic flexibility, thereby sustaining bioenergetic and redox homeostasis under stress [2,3]. In contrast, immune cells display metabolic vulnerability, as nutrient limitation and mitochondrial dysfunction impair their proliferation, effector function, and persistence [4]. Thus, mitochondrial amino acid metabolism not only supports tumor adaptation but also constrains antitumor immunity, highlighting a critical trade-off that shapes therapeutic responsiveness (Figure 1).

**Figure 2:**
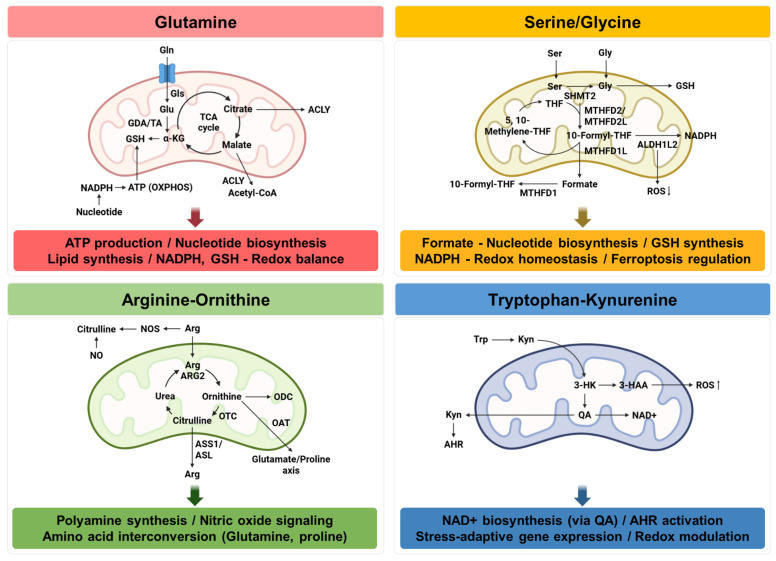
Mitochondrial amino acid metabolic pathways and their functional outputs in cancer. Major amino acid metabolic pathways converge within mitochondria to support bioenergetic, biosynthetic, and redox functions in cancer cells. Glutamine metabolism fuels the TCA cycle through conversion to α-ketoglutarate, supporting ATP production, nucleotide biosynthesis, lipid synthesis, and NADPH/GSH-mediated redox balance. Serine and glycine drive mitochondrial one-carbon metabolism via SHMT2 and MTHFD enzymes, generating formate for nucleotide synthesis and NADPH for redox homeostasis and ferroptosis regulation. The arginine–ornithine axis links the urea cycle to polyamine synthesis, nitric oxide signaling, and amino acid interconversion, contributing to cellular proliferation and metabolic flexibility. The tryptophan–kynurenine pathway produces metabolites such as quinolinic acid that support NAD^+^ biosynthesis and activate AHR-dependent stress-adaptive gene expression, while modulating mitochondrial redox status. Collectively, these pathways illustrate how amino acid metabolism integrates with mitochondrial function to sustain tumor growth and adaptation.

Given the growing interest in mitochondrial amino acid metabolism, this review focuses on four major amino acid axes that link tumor metabolic adaptation with immune regulation: glutamine-driven anaplerosis, serine/glycine-dependent one-carbon metabolism, arginine–ornithine metabolism, and tryptophan–kynurenine metabolism (Figure 2). These pathways differ in the strength and directness of their mitochondrial relevance. Glutamine and serine/glycine metabolism are supported by relatively direct mitochondrial mechanisms, whereas arginine/ornithine and tryptophan/kynurenine metabolism are included as mitochondria-associated axes with more limited and partly indirect mechanistic evidence. We discuss how these pathways are rewired in cancer cells, how they influence immune cell fate and function, and how their dysregulation promotes tumor progression and impairs antitumor immunity. We further consider how targeting these pathways may support the development of metabolic biomarkers and mitochondria-based strategies for cancer immunotherapy.

## 2. Amino Acid-Driven Mitochondrial Rewiring in the Tumor Microenvironment

### 2.1. Glutamine-Driven Anaplerosis and Mitochondrial Fitness

The most fundamental mitochondrial fate of glutamine is TCA cycle anaplerosis through the glutamine (Gln)–glutamate (Glu)–α-ketoglutarate (α-KG) axis [22,23]. In this pathway, glutamine is converted to glutamate by glutaminase (GLS). Glutamate is subsequently converted to α-KG through glutamate dehydrogenase (GDH) or transaminase (TA) reactions, thereby entering the TCA cycle [9,23]. α-KG replenishes TCA cycle intermediates and sustains flux toward malate and citrate generation, supporting OXPHOS-driven ATP production [24,25,26]. Beyond anaplerosis, glutamine contributes to mitochondrial redox homeostasis [9,27,28,29]. Glutamine-derived glutamate serves as a precursor for glutathione (GSH) synthesis. In addition, TCA-linked metabolism supports nicotinamide adenine dinucleotide phosphate (NADPH) generation [9,27,28,29]. Glutamine also serves as a nitrogen donor for purine and pyrimidine biosynthesis. Notably, *de novo* pyrimidine synthesis includes a mitochondrial step catalyzed by dihydroorotate dehydrogenase, which is located on the inner mitochondrial membrane [30]. However, these processes are mediated predominantly in the cytosol through amidotransferase reactions, including those catalyzed by phosphoribosyl pyrophosphate amidotransferase and carbamoyl phosphate synthetase II [31]. Under hypoxia or mitochondrial stress, α-KG may also undergo reductive carboxylation through isocitrate dehydrogenase 1/2 (IDH1/2), thereby sustaining citrate production [32,33,34,35]. Citrate generated in mitochondria can be exported to the cytosol, where it is converted to acetyl coenzyme A (acetyl-CoA) by ATP–citrate lyase (ACLY). This process supplies carbon for fatty acid and cholesterol biosynthesis [33,36,37,38,39]. Thus, mitochondrial glutamine metabolism primarily supports TCA cycle-driven ATP production and redox control, whereas its contributions to nucleotide and lipid biosynthesis are partly mediated through cytosolic processes.

Many cancers consume glutamine in large quantities to sustain rapid growth and proliferation [40,41,42]. The TME is characterized by hypoxia, acidosis, and nutrient deprivation [43,44,45]. To support glutamine uptake, tumor cells can utilize plasma-membrane transporters such as ASCT2 (SLC1A5). Members of the sodium-coupled neutral amino acid transporter (SNAT) family may also contribute, particularly under compensatory conditions [46,47]. These transporters have been known to mediate glutamine entry into the cytosol. Beyond tumor-intrinsic metabolism, glutamine uptake in the TME may also influence anti-tumor immunity. Recent evidence showed that tumor cells and conventional dendritic cells type 1 (cDC1s) compete for glutamine via *SLC38A2*-dependent transport. Glutamine availability in cDC1s is required for effective CD8^+^ T-cell activation and anti-tumor responses [48]. Interestingly, mitochondrial glutamine transport has recently been identified. The splice variant of *SLC1A5* mediates glutamine import into mitochondria through its N-terminal mitochondrial targeting sequence. Under hypoxic conditions, this variant is selectively induced by HIF-2α, and facilitates glutamine transport into mitochondria rather than the cytosol. Yoo et al. demonstrated that this pathway supports TCA cycle-associated ATP production and redox homeostasis [49]. In a subsequent study, pharmacologic inhibition of this variant in pancreatic cancer reduced ATP production and altered purine and glutathione metabolism, as shown by metabolomics profiling [50].

In immune cells, glutamine-dependent mitochondrial metabolism is linked not merely to nutrient supply but also to lineage-specific differentiation, inflammatory programming, and effector function. Inhibition of glutamine synthetase (GS), a cytosolic enzyme, can reprogram M2-like macrophages toward an M1-like phenotype. This shift is accompanied by decreased intracellular glutamine levels and attenuation of pro-inflammatory phenotype and pro-metastatic features [51,52]. Succinate, a mitochondrial TCA cycle intermediate, stabilizes HIF-1α and enhances IL-1β-driven inflammatory signaling, thereby promoting M1-like macrophage polarization [53]. Glutaminase, an inner-mitochondrial-membrane-associated enzyme, is required for NK-cell cytotoxicity. Its inhibition disrupts redox homeostasis and suppresses effector function [54]. Glutaminase-dependent metabolism promotes Th17 differentiation in CD4^+^ T cells, while constraining Th1 differentiation in CD4^+^ T cells and cytotoxic effector differentiation in CD8^+^ T cells [55]. Beyond this early effector programming, mitochondrial glutamine availability via glutaminase also influences the long-term fate of CD8^+^ T cells. This includes memory formation and exhaustion, indicating that glutamine flux contributes to effector-versus-memory fate decisions [55].

In summary, glutamine metabolism has one of the most direct mitochondrial links, particularly through mitochondrial glutaminase activity, α-KG-dependent TCA-cycle anaplerosis, OXPHOS, and redox regulation. By contrast, effects mediated by plasma membrane glutamine transporters, extracellular glutamine competition, or cytosolic glutamine synthetase are better interpreted as broader glutamine-dependent or mitochondria-associated mechanisms. These should not be considered strictly mitochondria-specific events (Table 1).

### 2.2. Serine/Glycine One-Carbon Metabolism and Redox Control

Mitochondrial one-carbon (1C) metabolism serves as a central hub that converts serine- and glycine-derived 1C units into formate, which is exported to the cytosol and nucleus to support nucleotide biosynthesis [56,57]. The 1C network is compartmentalized, with mitochondria generating 1C units and the cytosol utilizing them for purine and thymidylate synthesis [56,57]. Within mitochondria, the SHMT2–MTHFD2/MTHFD2L–MTHFD1L axis constitutes a major arm of the folate-mediated 1C circuit [57,58,59,60]. The formate is primarily generated through serine metabolism. Tetrahydrofolate (THF) accepts serine-derived 1C units, and serine hydroxymethyltransferase 2 (SHMT2) converts serine to glycine while generating 5,10-methylene-THF [61,62]. This intermediate is subsequently processed by MTHFD2/MTHFD2L to produce 10-formyl-THF or converted to formate by MTHFD1L for export to the cytosol [61,62]. Thus, in many proliferating cells, mitochondria serve as the principal source of 1C units, whereas the cytosol and nucleus are the major sites of 1C consumption for nucleotide biosynthesis [10,56,60,61]. In addition to nucleotide synthesis, mitochondrial 1C metabolism contributes to redox homeostasis. SHMT2-derived glycine supports GSH synthesis, and oxidation of 10-formyl-THF by aldehyde dehydrogenase 1 family member L2 (ALDH1L2) generates mitochondrial NADPH. These processes limit excessive ROS accumulation and preserve mitochondrial membrane potential under hypoxic or nutrient-restricted conditions in the TME [58,63]. Recent evidence further suggests that this redox regulation may influence susceptibility to ferroptotic cell death, as serine availability governs GSH synthesis and lipid peroxide detoxification [64]. Interestingly, SHMT2 also contributes to mitochondrial translation initiation by generating formylmethionyl-tRNA, indicating that its functions extend beyond nucleotide synthesis and redox homeostasis [59]. Collectively, mitochondrial serine/glycine-driven 1C metabolism primarily supports formate-dependent nucleotide biosynthesis and mitochondrial redox homeostasis, with additional implications for ferroptosis sensitivity.

In cancer cells, serine supply and mitochondrial 1C metabolism are frequently reinforced to sustain proliferation and stress adaptation [58,65]. Tumor cells enhance *de novo* serine synthesis and increase extracellular serine uptake, thereby expanding intracellular serine pools that supply mitochondrial 1C metabolism and downstream nucleotide biosynthesis [66,67]. In parallel, loss of tumor suppressor p53 further augments this pathway by relieving repression of *MTHFD2* expression, thereby increasing 1C flux, purine synthesis, and tumor proliferation [68]. Also, mitochondrial metabolic rewiring associated with high *MTHFD2* expression may alleviate the accumulation of toxic folate intermediates and promote tumor growth [68]. Under amino acid limitation or hypoxia, stress-responsive transcription factors, including ATF4 and HIF-1, upregulate mitochondrial 1C enzymes, such as SHMT2 and MTHFD2, thereby promoting metabolic adaptation [69,70,71]. Beyond its mitochondrial role, SHMT2 also exerts noncanonical functions. Liu and colleagues reported that, in colorectal cancer, SHMT2 has been detected in the cytosol and nucleus, where it interacts with β-catenin and TCF4 to inhibit β-catenin degradation, thereby promoting tumor growth and metastasis [72]. Collectively, mitochondrial serine/glycine 1C metabolism emerges as a multifunctional pathway that supports proliferation, stress adaptation, and oncogenic signaling.

This biosynthetic and redox-supporting role is also relevant to immune cells, although its functional outcome depends on activation state and lineage. Immune cells engage serine- and glycine-driven 1C metabolism in a context-dependent manner [2,73]. Genetic knockdown of *SHMT2* compromises post-activation survival and reduces the accumulation of activated T cells [74]. *MTHFD2* deficiency in naïve CD4^+^ T cells promotes differentiation toward regulatory T cells rather than Th17 cells, implying that MTHFD2 acts as a metabolic checkpoint controlling the Th17 regulatory T (Treg)-cell balance [74,75]. This mitochondrial 1C metabolic program aligns the biosynthetic demands of clonal expansion by maintaining the nucleotide pools required for rapid DNA replication [56,65,76]. Serine restriction impairs effector T-cell clonal expansion even under glucose-replete conditions, indicating that 1C units and glycine supply can become limiting [77]. Pharmacologic inhibition of SHMT1 and SHMT2 using RZ-2994 selectively suppresses T-cell proliferation without affecting early activation markers, suggesting that entry of serine-derived carbon into the 1C network functions as a biosynthetic checkpoint [77]. Importantly, the differentiation phenotype associated with *MTHFD2* loss can be rescued by formate supplementation or purine nucleobases, supporting a central role for 1C-dependent nucleotide supply [78]. In macrophages, serine metabolism regulates LPS-induced IL-1β expression primarily through GSH-mediated redox control rather than nucleotide biosynthesis [79,80]. Formate supplementation fails to restore IL-1β expression, whereas cell-permeable GSH rescues it, indicating that the serine/glycine axis sustains immune responses through redox regulation [80].

Taken together, the mitochondrial relevance of the serine/glycine axis is relatively strong because the SHMT2–MTHFD2/2L–MTHFD1L pathway directly generates formate and redox equivalents within mitochondria. Nevertheless, cytosolic serine synthesis, SHMT1–MTHFD1-mediated one-carbon reactions, nuclear/cytosolic nucleotide biosynthesis, and mitochondria-independent SHMT2 functions should be distinguished from direct mitochondrial one-carbon metabolism (Table 2).

### 2.3. Arginine–Ornithine Metabolism in Immune Regulation and Bioenergetic Adaptation

Mitochondrial arginine metabolism does not primarily function as a major carbon source for the TCA cycle or as a direct supplier of one-carbon metabolism. Instead, it serves as a regulatory interface with the urea-cycle and ornithine network, thereby coordinating nitrogen handling, amino acid interconversion, biosynthetic allocation, and immune regulatory programs [81,82]. Intracellular arginine is mainly processed through two principal pathways [81,82]. In the cytosol, nitric oxide synthase (NOS) converts arginine into citrulline and nitric oxide (NO), contributing to inflammatory signaling and microenvironmental modulation [81,82,83]. Alternatively, arginine is hydrolyzed by arginase enzymes—cytosolic arginase 1 (ARG1) or mitochondrial arginase 2 (ARG2)—to generate ornithine and urea. Ornithine can then be directed toward three major metabolic fates [81,82,84]. First, ornithine can re-enter the urea-cycle network through the mitochondrial ornithine transcarbamylase (OTC) reaction, generating citrulline. This citrulline is exported to the cytosol and reconverted to arginine through argininosuccinate synthase 1 (ASS1) and argininosuccinate lyase (ASL), with concomitant production of fumarate [84,85,86]. Second, ornithine can be converted by ornithine decarboxylase (ODC) into putrescine, initiating polyamine biosynthesis that proceeds to spermidine and spermine [87]. These polyamines interact with nucleic acids and components of the translational machinery to promote a cellular state favorable for proliferation and biosynthetic expansion [88,89]. Finally, within mitochondria, ornithine aminotransferase (OAT) converts ornithine into intermediates linked to the glutamate/proline axis. This process couples arginine metabolism to broader amino acid interconversion networks [90]. Beyond nitrogen disposal, arginine metabolism can indirectly influence mitochondrial bioenergetics. Urea-cycle-associated reactions generate fumarate, which can re-enter the TCA cycle and contribute to anaplerotic flux under specific metabolic conditions. In addition, OAT-mediated conversion of ornithine into glutamate provides a metabolic link to the glutamine–TCA axis, suggesting that arginine metabolism may support mitochondrial function in a context-dependent manner [85,91]. Thus, rather than acting as a primary carbon fuel, arginine conditionally supports mitochondrial metabolism through its integration with TCA cycle intermediates and amino acid interconversion pathways.

In cancer cells, arginine metabolism is frequently rewired to support proliferation and adaptation to nutrient stress. Many cancers downregulate urea-cycle enzymes such as ASS1, thereby limiting citrulline-based arginine recycling and increasing dependence on extracellular arginine [92]. In *KRAS*-mutant non-small cell lung cancer, repression of *ASS1* redirects aspartate toward *de novo* pyrimidine synthesis to support DNA replication [93]. In hepatocellular carcinoma (HCC), arginine can accumulate despite reduced expression of ASS1 and other urea-cycle genes, likely owing to increased uptake and altered metabolic allocation [94]. Mossmann et al. further showed that accumulated arginine binds RBM39 to promote metabolic reprogramming, including induction of *ASNS* and an asparagine–arginine antiporter, thereby reinforcing tumor-supportive arginine homeostasis [94]. In pancreatic ductal adenocarcinoma (PDAC), tumors exhibit increased reliance on OAT-mediated ornithine production from glutamine to sustain polyamine biosynthesis, thereby circumventing extracellular arginine limitation. Given that arginine levels in tumor interstitial fluid are frequently lower than in normal tissues, PDAC appears to circumvent extracellular arginine limitation by generating ornithine internally via glutamine metabolism [95,96,97]. These cancer-type-specific strategies indicate that arginine–ornithine metabolism reflects an integrated rewiring program that balances arginine availability with selective routing of ornithine toward biosynthesis and metabolic pathways.

Within the TME, arginine metabolism also plays a central role in immune suppression. Tumor-associated myeloid cells, including myeloid-derived suppressor cells and tumor-associated macrophages, frequently express high levels of ARG1, leading to extra-cellular arginine depletion. This reduction in arginine availability impairs T-cell proliferation, cytokine production, and survival, thereby limiting effective antitumor immune responses. Thus, arginine metabolism represents a key metabolic checkpoint that links tumor-driven nutrient competition to immune dysfunction. While extracellular arginine depletion suppresses immune responses, intracellular arginine metabolism within immune cells also plays a critical role in determining their functional state. Arginine availability and ornithine-derived metabolites also shape immune-cell fitness and inflammatory programs. In immune cells, arginine metabolism is increasingly recognized as an immunoregulatory network rather than merely a biosynthetic pathway. It governs metabolic fitness and differentiation in T cells and influences pro-inflammatory versus immunoregulatory responses in macrophages and dendritic cells [52,98]. In CD8^+^ T cells, arginase activity is largely attributable to mitochondrial ARG2 rather than ARG1, linking mitochondrial metabolic state to nitrogen disposal [98]. Cytosolic arginine can be transported into mitochondria and hydrolyzed by ARG2 to generate urea and ornithine, thereby contributing to ammonia detoxification and supporting memory T-cell development [99]. Conversely, Zhang et al. demonstrated that ammonia derived from glutamine catabolism may accumulate during rapid effector CD8^+^ T-cell proliferation. Excess accumulation disrupts lysosomal and mitochondrial integrity and induces cell death [100]. These observations underscore that nitrogen-handling capacity is a critical determinant of immune-cell viability and differentiation. Beyond nitrogen detoxification, ornithine-polyamine metabolism also plays immunoregulatory role. Polyamine synthesis supports CD4^+^ T-cell differentiation, whereas loss of *ODC1* disrupts lineage-specific polarization [101]. Mitochondrial ARG2 also participates in anti-inflammatory feedback circuits. IL-10-mediated signal transducer and activator of transcription 3 (STAT3) signaling increases *ARG2* expression via suppression of miR-155. Elevated *ARG2* enhances succinate dehydrogenase (SDH, complex II) activity, reduces succinate accumulation, and attenuates IL-1β-associated inflammatory signaling [102,103]. Arginine availability is further coupled to signaling through canonical amino acid-sensing systems, including CASTOR1 and SLC38A9. In activated T cells, increased extracellular L-arginine promotes oxidative metabolism, a memory-like phenotype, and survival, potentially through regulatory factors such as BAZ1B, PSIP1, and TSN [2,15,104].

Taken together, the arginine–ornithine axis is linked to mitochondria through specific metabolic nodes rather than through the entire pathway. ARG2, OTC, OAT, and fumarate-linked anaplerotic return provide direct mitochondrial links, whereas NOS signaling, ARG1 activity, ASS1/ASL-dependent recycling, ODC-driven polyamine synthesis, and extracellular arginine sensing are broader cytosolic or systemic mechanisms (Table 3).

### 2.4. Tryptophan–Kynurenine Metabolism in Immune Suppression and Mitochondrial Dysfunction

The tryptophan–kynurenine (Trp–Kyn) pathway represents the dominant route of tryptophan catabolism, accounting for approximately 95% of systemic Trp degradation [105,106]. Entry into this pathway is initiated by indoleamine 2,3-dioxygenase 1/2 (IDO1/IDO2) or tryptophan 2,3-dioxygenase (TDO; predominantly TDO2), which convert Trp to N-formylkynurenine, subsequently processed into Kyn and downstream metabolites [106,107]. These metabolites, including 3-hydroxykynurenine (3-HK), 3-hydroxyanthranilic acid (3-HAA), and QA, contribute to NAD^+^ biosynthesis and regulate redox homeostasis [105,106,108]. Kyn and related indole derivatives function as endogenous ligands for the aryl hydrocarbon receptor (AHR), a transcription factor that coordinates stress-adaptation and metabolic programs [109,110]. In addition, components of the Trp–Kyn pathway exhibit direct or indirect links to mitochondrial function. Kynurenine 3-monooxygenase (KMO) localizes to the outer mitochondrial membrane and is associated with redox and energy metabolism [106,111,112]. Importantly, metabolites such as 3-HK increase oxidative stress and impair TCA cycle activity through inhibition of ROS-sensitive enzymes [113]. Collectively, the Trp–Kyn axis integrates AHR-dependent transcriptional regulation with mitochondrial redox control.

In cancer cells, tryptophan metabolism contributes to tumor progression through both transcriptional and metabolic mechanisms [107,114]. Activation of AHR by Kyn induces gene expression programs associated with proliferation, invasion, and metastasis [107,115]. In glioma, increased intratumoral IDO1 and TDO activity correlates with elevated Kyn release, AHR activation, and induction of aquaporin-4 expression, which has been implicated in enhanced tumor-cell motility [116]. In HCC, Kyn-driven AHR signaling promotes EMT via Slug (*SNAI2*)-mediated E-cadherin suppression, although links to vimentin and MMP9 remain unclear [117,118]. Evidence for tumor-promoting functions of IDO1 has been provided in colorectal cancer and colitis-associated tumorigenesis models [119,120]. Pharmacological inhibition or genetic deletion of *IDO1* reduced tumor burden and epithelial proliferation, with these effects persisting in recombination-activating gene 1 (*RAG1*)-deficient mice, indicating a partially T-cell-independent mechanism. Mechanistically, supplementation with Kyn or QA restores β-catenin activity and proliferative capacity in IDO1-inhibited contexts [119]. Furthermore, in severe combined immunodeficiency (SCID)/beige mice lacking T-, B-, and NK cells, IDO1-positive tumors grow more rapidly than IDO1-negative tumors, supporting a cancer cell-intrinsic proliferative role [120]. Tryptophan metabolism is also closely linked to NAD^+^ synthesis, which may influence tumor aggressiveness and therapeutic response [111,112,121,122,123]. In murine liver models, suppression of Trp-derived *de novo* NAD^+^ synthesis increases DNA damage accumulation and enhances hepatocarcinogenesis [122]. In human glioblastoma, Trp-derived NAD^+^ production has been associated with resistance to oxidative stress induced by radiotherapy and chemotherapy [112]. IDO1 activity may further augment NAD^+^ availability and DNA repair capacity, contributing to resistance to poly (ADP-ribose) polymerase (PARP) inhibitors, radiotherapy, and cisplatin [123].

Beyond these tumor-intrinsic effects, Trp catabolism also promotes immune evasion through local Trp depletion and Kyn-derived signaling [124,125]. Through local tryptophan depletion and the accumulation of kynurenine, this pathway suppresses antitumor immunity and alters immune-cell differentiation and function [124,125]. Clinical observations of reduced circulating Trp, increased intratumoral Kyn levels, and elevated Kyn-to-Trp ratios support the notion that Trp depletion-driven immunosuppression is most pronounced locally within tumors [126]. In tumor-infiltrating antigen-presenting cells and tumor-associated macrophages, IDO1 induced by inflammatory cytokines such as IFN-γ establishes a Trp-depleted niche that impairs effector T-cell proliferation and function [127,128]. In tumor-associated macrophages (TAM), AHR signaling reinforces immunosuppressive polarization, including induction of IL-10, ARG1, and PD-L1 [129,130]. In CD8^+^ T cells, AHR activation promotes an exhaustion-associated program characterized by increased inhibitory receptors (PD-1, CTLA-4, LAG3) and transcription factors such as TOX and NR4A family members, accompanied by reduced cytotoxic effector function [131,132]. At the same time, cytotoxic effector genes including *IFN-γ*, granzyme B (*GZMB*), and perforin (*PRF1*) decline, reflecting functional remodeling toward exhaustion [68,132]. In dendritic cells, the Trp–Kyn axis promotes tolerogenic reprogramming through both tryptophan depletion and kynurenine–AHR signaling, including noncanonical NF-κB activation [133]. Trp depletion also activates the amino-acid-sensing kinase GCN2, thereby promoting T-cell anergy and regulatory T-cell differentiation [134,135]. In parallel, IDO-expressing myeloid-derived suppressor cells can further reinforce immunosuppression in cancer settings [136]. In NK cells, kynurenine accumulation suppresses antitumor activity by impairing activating receptor expression, including NKp46 and NKG2D, and by promoting ROS-associated loss of NK-cell viability [137,138]. In triple-negative breast cancer and NK cell co-culture systems, IDO-1 also reduces NK-cell cytotoxicity through HLA-G upregulation in tumor cells [139].

Taken together, compared with glutamine and serine/glycine metabolism, the Trp–Kyn axis is less directly linked to mitochondrial metabolism. Its mitochondrial relevance is mainly supported by KMO localization at the outer mitochondrial membrane, 3-HK-associated oxidative stress and TCA-cycle perturbation, and QA-dependent NAD^+^ biosynthesis. In contrast, IDO1/TDO-mediated Trp depletion, Kyn–AHR signaling, GCN2 activation, and immune suppression largely reflect cytosolic, nuclear, or extracellular mechanisms rather than direct mitochondrial regulation (Table 4).

## 3. Implications for Cancer Immunotherapy

In applying the regulation of amino acid metabolism, including the glutamine, serine/glycine, arginine/ornithine, and tryptophan/kynurenine axes, to cancer immunotherapy, the central challenge is to exploit tumor-specific metabolic vulnerabilities while preserving antitumor immune function [140,141]. To provide a structured perspective, therapeutic strategies are evaluated based on tumor selectivity, immune-cell risk, mechanistic rationale, translational stage, and key limitations.

Targeting glutamine metabolism has been widely pursued due to its central role in mitochondrial bioenergetics and redox homeostasis. Broad glutamine antagonists such as 6-Diazo-5-oxo-L-norleucine (DON) exhibit strong antitumor activity but lack tumor selectivity, resulting in systemic toxicity, particularly in the gastrointestinal tract [142]. The prodrug JHU-083 improves tumor selectivity and demonstrates both tumor suppression and immune-modulatory effects, including reduced myeloid-derived suppressor cell accumulation and enhanced CD8^+^ T-cell responses [143]. More selective strategies include GLS1 inhibitor CB-839, which has demonstrated *in vivo* efficacy and has progressed to clinical evaluation [144,145]. However, its effects are context-dependent, as glutaminase activity also regulates T-cell differentiation, creating potential immune-cell risk [55]. Targeting mitochondrial glutamine transport through *SLC1A5* splice variants offers an additional level of selectivity by directly impairing tumor mitochondrial metabolism. Notably, such inhibition can induce PD-L1 upregulation, thereby providing a mechanistic rationale for combination strategies with immune checkpoint blockade [50]. Nevertheless, the expression and function of this variant in immune cells remain incompletely defined, highlighting a key limitation in predicting tumor selectivity [50].

Targeting one-carbon metabolism is supported by a clear mechanistic rationale, as this pathway sustains nucleotide biosynthesis and redox balance. SHMT inhibitors such as SHIN1 and SHIN2 suppress one-carbon flux, with SHIN2 showing *in vivo* efficacy and synergy with antifolate therapy [146]. Because SHMT1 and SHMT2 can function redundantly, simultaneous inhibition is required to prevent metabolic compensation [147,148]. Sertraline, originally developed as an antidepressant, has been repurposed as an SHMT inhibitor. When combined with the putative mitochondrial inhibitor artemether, it demonstrated *in vivo* efficacy in breast cancer xenograft models [149]. Tumor selectivity may be achievable through differential expression of mitochondrial one-carbon enzymes, including MTHFD2, which is elevated in tumors but low in normal tissues [65]. However, this pathway also supports T-cell proliferation and effector function, indicating a significant immune-cell risk. Indeed, inhibition of MTHFD2 can alter CD4^+^ T-cell differentiation and impair proliferative expansion, although some metabolic defects can be partially rescued by formate or purine supplementation [150,151]. These findings suggest that therapeutic benefit may depend on temporally controlled modulation rather than continuous inhibition. For example, *ex vivo* manipulation during CAR-T cell expansion provides a controlled setting to optimize metabolic conditions while minimizing systemic immune effects [152]. Nonetheless, clinical translation remains limited by metabolic redundancy, interpatient variability, and the lack of real-time biomarkers.

Arginine-targeting strategies offer a more defined framework for tumor selectivity through biomarker-guided approaches. Tumors with low *ASS1* expression depend on extracellular arginine, supporting the use of arginine-depleting agents such as ADI-PEG20 [153]. Clinical studies combining ADI-PEG20 with anti-PD-1 therapy have reported acceptable safety profiles and evidence of immune modulation, indicating translational potential [154]. At the same time, arginine metabolism directly influences immune function. Myeloid-derived arginase activity can deplete arginine within the TME, suppressing T-cell responses. This provides a rationale for arginase inhibitors such as INCB001158, which aim to restore local arginine availability and enhance checkpoint blockade efficacy [154,155,156]. Beyond pharmacologic approaches, adaptive immune cell strategies have been developed to enhance arginine resilience. These include *ex vivo* conditioning through controlled L-arginine and engineering CAR-T cells with *ASS1*- or *OTC* to enable arginine resynthesis [154,157,158]. Furthermore, targeting downstream polyamine metabolism may reinforce immunosuppressive myeloid programs; polyamine-depleting strategies such as α-difluoromethylornithine (DFMO) combined with the polyamine transport inhibitor AMXT 1501 are under investigation in immunotherapy combinations [159,160]. Complementary approaches, including therapeutic vaccination targeting immunosuppressive mediators such as ARG1, further aim to reprogram suppressive myeloid compartments [161,162]. Collectively, arginine-targeting approaches demonstrate relatively high tumor selectivity but require careful balancing of immune modulation and metabolic compensation.

Within the Trp–Kyn axis, clinical trials of IDO1 inhibitors point to a potential limitation of single-enzyme targeting in complex immunometabolic networks. Epacadostat plus pembrolizumab was well tolerated and showed encouraging antitumor activity in the phase I/II ECHO-202/KEYNOTE-037 trial, but this signal was not confirmed in subsequent randomized phase III studies [163]. In ECHO-301/KEYNOTE-252, epacadostat plus pembrolizumab failed to improve progression-free or overall survival in unresectable or metastatic melanoma [164]. Similarly, ECHO-307/KEYNOTE-672 showed a similar objective response rate and safety profile between epacadostat plus pembrolizumab and placebo plus pembrolizumab in cisplatin-ineligible urothelial carcinoma [165]. Together, these findings suggest that selective IDO1 inhibition alone may be insufficient to control the broader Trp–Kyn network, supporting broader strategies such as dual IDO1/TDO2 targeting, AHR-directed approaches, biomarker-guided patient selection, and immune-reprogramming strategies [105,166]. This has shifted attention toward broader immunometabolic strategies [167]. In this context, the IO102/IO103 vaccine represents an alternative approach aimed at immune reprogramming rather than direct metabolic blockade, and its combination with anti-PD-1 therapy has shown encouraging early clinical activity [168,169].

**Figure 3:**
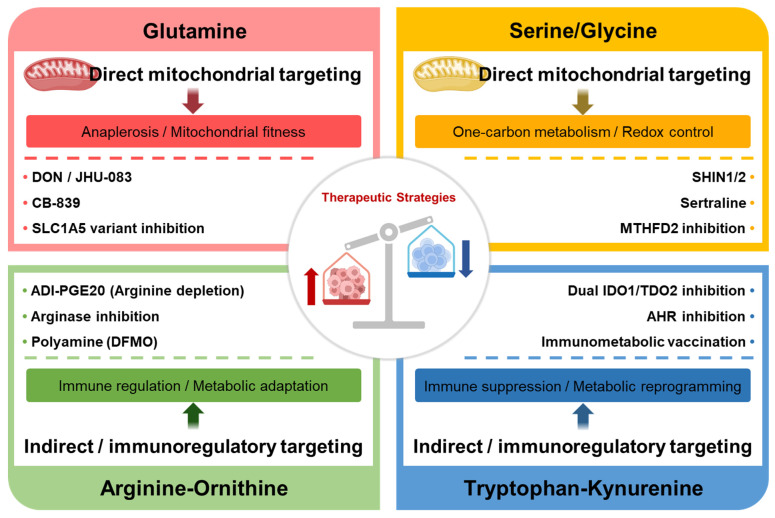
Therapeutic strategies targeting amino acid metabolism and their mitochondrial relevance in cancer immunotherapy. Glutamine, serine/glycine, arginine/ornithine, and tryptophan/kynurenine metabolic pathways support tumor progression and immune regulation through distinct mechanisms. While glutamine and serine/glycine axes directly sustain mitochondrial bioenergetics and redox homeostasis, arginine and tryptophan pathways primarily modulate immune responses and metabolic signaling. Targeting these pathways offers opportunities to enhance antitumor immunity while exploiting tumor-specific metabolic vulnerabilities. Dashed lines separate representative therapeutic strategies from their corresponding metabolic or immunoregulatory effects; different colors denote distinct amino acid pathways; arrows indicate the direction of therapeutic modulation toward tumor metabolic suppression or immune restoration.

Collectively, amino acid-targeted therapies should be assessed by tumor selectivity, immune-cell effects, mechanism of action, translational status, and resistance barriers. Tumor selectivity may be enhanced by tumor-directed delivery of JHU-083, inhibition of tumor-enriched mitochondrial one-carbon enzymes such as MTHFD2, or selection of ASS1-deficient tumors for ADI-PEG20. However, because these pathways also sustain immune-cell function, such strategies may affect NK-cell cytotoxicity, T-cell fate decisions, activated T-cell proliferation, and arginine-dependent T-cell fitness. Mechanistically, they act by limiting glutamine utilization, suppressing mitochondrial one-carbon metabolism, modulating arginine availability, or disrupting Trp–Kyn–AHR signaling. Their development spans preclinical strategies, including JHU-083, SHIN2, and sertraline plus artemether, and clinically evaluated agents, including CB-839, ADI-PEG20, arginase inhibitors, IDO1 inhibitors, and IO102/IO103 vaccination. Remaining barriers include systemic toxicity, high dose requirements, compensatory rewiring, pathway redundancy, tumor heterogeneity, and insufficient biomarker-guided patient selection (Table 5) (Figure 3).

## 4. Conclusions

The four major amino acid metabolic axes converge on mitochondrial rewiring in both tumor and immune cells, thereby shaping tumor progression and antitumor immunity in parallel. However, the therapeutic evidence base remains uneven across these axes. Their relevance extends beyond simply restricting tumor metabolism, because the key question is whether a given intervention can exploit tumor vulnerabilities while preserving or enhancing immune effector function. Preclinical studies targeting glutamine metabolism or mitochondrial one-carbon metabolism support this possibility. For example, the DON prodrug JHU-083 reduced tumor burden, decreased immunosuppressive myeloid-derived suppressor cells, and enhanced CD8^+^ T cell-mediated antitumor responses [170], whereas sertraline combined with artemether showed *in vivo* efficacy in a breast cancer xenograft model [149]. These findings suggest that selected amino acid-directed interventions may simultaneously impair tumor fitness and favorably modulate the immune microenvironment.

Nevertheless, clinical experience indicates that single-enzyme targeting of amino acid metabolism has generally produced limited therapeutic benefit, although arginine-depleting strategies demonstrate that meaningful clinical activity can be achieved in tumors with defined arginine dependence, such as *ASS1*-deficient or arginine-auxotrophic tumors. The modest outcomes observed with GLS1 inhibition and IDO1 inhibition suggest that blockade of a single metabolic or immunometabolic enzyme is often insufficient to overcome the adaptive, redundant, and compartment-specific nature of tumor metabolism and immune suppression. In addition, tumors may escape metabolic intervention through compensatory pathway activation, metabolic plasticity, or reliance on alternative nutrient sources, whereas systemic or non-selective metabolic inhibition may impair immune-cell function and narrow the therapeutic window. Direct mitochondrial targeting also remains difficult to translate, as illustrated by the limitations of biguanides such as phenformin and metformin [5,171].

These considerations highlight the need to move beyond broad mitochondrial inhibition or simple single-enzyme blockade. Combination strategies with immune checkpoint inhibitors remain particularly promising, especially when metabolic intervention can relieve nutrient competition, myeloid suppression, or mitochondrial insufficiency within the TME while checkpoint blockade restores antitumor immunity constrained by inhibitory receptor signaling. In parallel, dual metabolic targeting without immune checkpoint blockade deserves further investigation. For example, combining inhibition of mitochondria-linked glutamine metabolism or mitochondrial one-carbon metabolism with blockade of the IDO–kynurenine axis could, in principle, weaken tumor mitochondrial metabolism, nucleotide synthesis, redox balance, and anabolic fitness while reducing tryptophan catabolism-associated immunosuppressive signaling. However, this concept remains experimental and requires careful validation to determine whether it can selectively impair tumor fitness without compromising metabolically active immune effector cells. Future progress will therefore depend on biomarker systems that integrate tumor metabolic states, metabolite availability within the TME, and mitochondrial functional indices in immune cells, thereby supporting patient stratification, treatment timing, and rational combination strategies that preserve or enhance antitumor immune function.

## Figures and Tables

**Table 1:** Glutamine-driven mitochondrial rewiring in tumor cells and immune cells within the TME.

Cell Type		Glutamine-Driven Mitochondrial Rewiring	Mitochondrial Relevance	Functional Outcome	References
Cancer cells	Many cancer cells	Mitochondrial glutamine transport via the SLC1A5 variant supports TCA cycle anaplerosis, ATP production, and redox balance	Direct	Supports tumor survival and metabolic plasticity under stress	[22,23,24,25,26,27,28,29,49,50]
Immune cells	Macrophages	Succinate–HIF-1α–IL-1β signaling influence inflammatory programming	Indirect	Promotes macrophage polarization toward a more M1-like/pro-inflammatory state	[51,52,53]
	Natural Killer cells	Glutaminase-dependent metabolism maintains redox homeostasis	Direct	Supports NK-cell cytotoxic effector function	[54]
	CD4^+^ T cells	Glutamine metabolism regulates CD4^+^ T-cell lineage commitment	Direct	Impairs Th17 differentiation but may promote Th1 differentiation	[55]
	CD8^+^ T cells	Glutaminase-dependent mitochondrial metabolism regulates early effector programming and long-term cell fate	Direct	Influences effector function, memory formation, and exhaustion	[55]

**Table 2:** Serine/glycine-driven mitochondrial rewiring in tumor cells and immune cells within the TME.

Cell Type		Serine/Glycine-Driven Mitochondrial Rewiring	Mitochondrial Relevance	Functional Outcome	References
Cancer cells	Multiple cancers	Increased level of serine and glycine for mitochondrial 1C metabolism	Indirect	Enhanced 1C metabolism and nucleotide biosynthesis	[56,57,60,62,66,67]
		Activation of the mitochondrial SHMT2–MTHFD2 axis supports 1C metabolism, formate production, and NADPH generation	Direct	Supports nucleotide biosynthesis, tumor proliferation, and redox homeostasis	[57,58,59,60,61,62,63]
		Stress adaptation via ATF4- and HIF1α-mediated induction of *SHMT2/MTHFD2*	Direct	Promotes metabolic adaptation under nutrient and hypoxic stress conditions	[69,70,71]
	Colorectal cancer	SHMT2 activation	Direct	Inhibition β-catenin stabilization and pro-metastatic signaling	[72]
Immune cells	T cells	Activation of the mitochondrial SHMT2–MTHFD2/2L–MTHFD1L axis	Direct	Support of T-cell proliferation and clonal expansion	[56,65,74,76,77]
		MTHFD2 acts as a metabolic checkpoint	Direct	Controls T-cell lineage commitment and immune function	[74,75]
	Macrophages	GSH-dependent redox maintenance	Direct	Macrophage immune response including IL-1β	[79,80]

**Table 3:** Arginine/ornithine-driven mitochondrial rewiring in tumor cells and immune cells within the TME.

Cell Type		Arginine/Ornithine-Driven Mitochondrial Rewiring	Mitochondrial Relevance	Functional Outcome	References
Cancer cells	Non-small lung cancer	Downregulation of urea-cycle enzymes, including ASS1	Indirect	Impaired citrulline-to- arginine flux and redirection of aspartate toward pyrimidine synthesis	[93]
	Hepatocellular carcinoma	Reduced urea-cycle activityalters arginine utilization and limits polyamine synthesis	Indirect	Reprograms arginine metabolism and limits polyamine synthesis	[94]
	Hepatocellular carcinoma	Arginine accumulation associated with RBM39 signaling reinforces a high-arginine metabolic state	Indirect	Reinforces a high-arginine metabolic state via *ASNS* upregulation	[94]
	PDAC	OAT-mediated *de novo* ornithine production from glutamine sustains intracellular ornithine supply	Direct	Enables adaptation to arginine limitation and sustains polyamine synthesis	[95,96,97]
Immune cells	T cells	ARG2-mediated mitochondrial arginine metabolism regulates urea and ornithine production	Direct	Promotes CD8^+^ memory T-cell formation and metabolic fitness	[98,99,100]
	CD4^+^ T cells	Polyamine synthesis via *ODC1* supports lineage-specific differentiation programs	Indirect	Promotes helper T-cell differentiation and subset stability	[101]
	Activated T cells	T-cell-specific arginine sensing by *BAZ1B*, *PSIP1*, and *TSN*	Indirect	Promotion of oxidative metabolism, a memory-like phenotype, and survival	[2,15,104]
	Macrophages	ARG2-mediated regulation of mitochondrial metabolism influences succinate turnover	Indirect	Suppresses IL-1β-mediated inflammatory signaling via succinate regulation	[102,103]

**Table 4:** Tryptophan–kynurenine-driven mitochondrial rewiring in tumor cells and immune cells within the TME.

Cell Type		Tryptophan–Kynurenine-Driven Mitochondrial Rewiring	Mitochondrial Relevance	Functional Outcome	References
Cancer cells	Glioma	Increased IDO1/TDO activity enhances kynurenine production and AHR activation	Indirect	*AQP4* induction and increased cell motility	[116]
	Hepatocellular carcinoma	Kynurenine-driven AHR signaling activates EMT-associated gene expression	Indirect	Upregulation of EMT-related genes, with reduced E-cadherin	[117,118]
	Colorectal cancer	IDO1-mediated tryptophan catabolism generates Kyn and QA, supporting AHR activation and NAD^+^ biosynthesis	Indirect	Enhances tumor proliferation and β-catenin–dependent growth programs	[119,120]
	Multiple cancers	Trp-derived metabolites support de novo NAD^+^ biosynthesis	Indirect	Promotes metabolic adaptation and resistance to oxidative stress and therapy	[111,112,121,122,123]
Immune cells	CD8^+^ T cells	Kyn-mediated AHR activation induces inhibitory receptor expression and exhaustion-associated transcription factors	Indirect	Promotes T-cell exhaustion	[131,132]
	Dendritic cells	IDO1-mediated Trp catabolism and Kyn–AHR signaling establish a positive feedback loop	Indirect	Promotes tolerogenic phenotype and suppresses T-cell activation	[133]
	CD4^+^ T cells	Tryptophan depletion activates GCN2 signaling	Indirect	Induces T-cell anergy and promotes Treg differentiation	[134,135]
	Natural killer cells	Tryptophan depletion and Kyn accumulation impair metabolic function	Indirect	Reduces cytotoxicity and cytokine production	[137,138,139]

**Table 5:** Therapeutic strategies targeting amino acid metabolism in cancer immunotherapy.

Amino Acid	Target		Mitochondrial Relevance	Mechanism	Limitation	References
Glutamine	Cancer	DON/ JHU-083	Indirect	Broad inhibition of glutamine metabolism	Systemic toxicity (DON), selectivity required	[142,143]
		GLS inhibitor (CB-839)	Direct	Inhibits glutaminase and TCA anaplerosis	Variable efficacy, high dose requirement	[144,145]
		*SLC1A5* variant inhibition	Direct	Blocks mitochondrial glutamine transport	Immune-cell selectivity unclear	[50]
Serine/ Glycine	Cancer	SHIN1/ SHIN2	Direct	Dual inhibition of SHMT1/2 and 1C metabolism	Compensation viaparallel pathways	[146,147,148]
		Sertraline + artemether	Direct	Repurposed SHMT inhibition + mitochondrial targeting	Limited clinical validation	[149]
	Cancer and T cell	*MTHFD2* inhibition	Direct	Targets mitochondrial one-carbon metabolism	Also affects proliferating T cells	[65,150,151]
Arginine/ ornithine	Cancer	ADI-PEG20	Indirect	Arginine depletion in *ASS1*-deficient tumors	Biomarker-dependent efficacy	[153,154]
		Arginase inhibitors	Indirect	Restores arginine availability	Tumor heterogeneity	[154,155,156]
		Polyamine blockade	Indirect	Inhibits polyamine synthesis/transport	Limited data	[159,160]
	CAR T cells	Engineered T cells	Indirect	Enhances arginine resynthesis	Translational complexity	[157,158]
	TAMs and T cell	ARG1 vaccination	Indirect	Synergize with Anti-PD-1 therapy	Dependence on effective ARG1-specific T-cell priming	[161,162]
Tryptophan/ kynurenine	Cancer	IDO1 inhibitors	Indirect	Blocks tryptophan catabolism	Redundant pathways	[106,169]
	Immune cells	IO102/IO103 vaccine	Indirect	Targets IDO1/TDO expressing cells	Limited validation	[168,169]
	Immune cells	AHR targeting	Indirect	Disrupts Kyn-mediated signaling	Early-stage development	[105,166]

## Data Availability

No new data were created or analyzed in this study. Data sharing is not applicable to this article.
